# Effects of a Virtual Reality Game on Children’s Anxiety During Dental Procedures (VR-TOOTH): Protocol for a Pilot Randomized Controlled Trial

**DOI:** 10.2196/49956

**Published:** 2023-11-10

**Authors:** Wenjia Wu, Sylvie Le May, Nicole Hung, Olivier Fortin, Christine Genest, Maxime Francoeur, Estelle Guingo, Kate St-Arneault, Annie Sylfra, An Kateri Vu, Janick Carmel, Laurence Lessard, Stephany Cara-Slavich, Katheryn De Koven, Julie Paquette, Hunter Hoffman, Marie-Eve Asselin

**Affiliations:** 1 Department of Dental Medicine Centre Hospitalier Universitaire Sainte-Justine Montréal, QC Canada; 2 Research Center Centre Hospitalier Universitaire Sainte-Justine Montreal, QC Canada; 3 Faculty of Nursing University of Montreal Montreal, QC Canada; 4 Faculty of Medicine University of Montreal Montreal, QC Canada; 5 Trauma Studies Centre Le Centre intégré universitaire de santé et de services sociaux de l’Est-de-l’Île de Montréal Montreal, QC Canada; 6 Université du Québec en Abitibi-Témiscamingue Quebec, QC Canada; 7 Department of Anesthesia Centre Hospitalier Universitaire St-Justine Hospital Montreal, QC Canada; 8 University of Washington Seattle, WA United States

**Keywords:** virtual reality, pediatrics, children, dentistry, procedures, fear, anxiety, child, pediatric, dentist, dental, tooth, teeth, oral, anxious, immersion, immersive, RCT, randomized, controlled trial

## Abstract

**Background:**

Dental fear and anxiety (DFA) is a condition that affects approximately one-quarter of children and adolescents. It is a significant cause for pediatric patients to avoid dental care later in adulthood. Lack of patient cooperation due to DFA can create an environment of stress, often obligating dentists to end appointments prematurely and consider alternative pharmacological treatment options. Virtual reality (VR) use during dental care, providing an immersive experience through sensory stimuli, is potentially an additional nonpharmacologic tool to better manage DFA in children with special health care needs (SHCN) undergoing dental procedures.

**Objective:**

This pilot study aims to assess the feasibility and acceptability of VR immersion as a tool to reduce DFA in pediatric special needs patients undergoing dental procedures. The study also aims to gain insight on parent and health care provider perspectives on the use of VR during dental appointments.

**Methods:**

This pilot randomized controlled trial study will follow a parallel design including 2 groups: a control group (clinic’s standard care using a wall TV) and an experimental group (using a VR game). We will randomize 20 participants to either group. Recruitment will be carried out at the dental clinic of the Centre Hospitalier Universitaire Sainte-Justine, a tertiary-quaternary care center that mostly serves pediatric patients with SHCN. The primary outcome will be patient recruitment rates and completion rates of planned procedures. DFA in children will be assessed using both an observation-based proxy assessment with the Venham Anxiety and Behavior Rating Scale and physiological assessments using parameters such as change in heart rate and levels of salivary alpha-amylase as a stress biomarker before and 10 minutes after the procedure. Sociodemographic characteristics, measures of the levels of parent and health care professional satisfaction, occurrence of side effects, and any deviation from normal procedure length will also be collected. Descriptive statistics, nonparametric tests, and effect sizes will be used for demographic and clinical variables and to present parent and health care professional satisfaction levels as well as procedural time.

**Results:**

This study will be conducted from May 2023 to May 2024, with results expected to be available in December 2024.

**Conclusions:**

The pilot study will provide insight on the feasibility and acceptability of VR use in clinical dentistry to reduce DFA for pediatric patients with SHCN. This study will guide future research on VR use in pediatric dentistry and can serve as a framework for a larger randomized clinical trial.

**Trial Registration:**

ClinicalTrials.gov NCT05898100; https://classic.clinicaltrials.gov/ct2/show/NCT05898100

**International Registered Report Identifier (IRRID):**

DERR1-10.2196/49956

## Introduction

Dental fear and anxiety (DFA) is a condition that affects approximately 13.3% to 29.3% of children and adolescents and is a significant cause of patients avoiding dental care [1,2]. In children, DFA is also associated with a lower oral health–related quality of life [3]. Although the etiology of DFA is multifactorial, often stemming from both exogenous and endogenous sources, a previous traumatic dental experience is the most predictive factor for DFA [4,5]. A study by Ten Berge et al [6] stated that the majority of DFA experienced by adults stems from poor dental experiences as children. These findings highlight the importance of keeping each dental experience a positive one for pediatric patients. Short-term distress during appointments that is not managed properly can accumulate into poor dental experiences and, in turn, reinforce DFA into adulthood [7]. The long-term effects of these poor dental experiences as children can lead patients to avoid seeking proper dental care in the future [8].

Dental patients with special health care needs (SHCN) are defined as patients requiring additional time and special consideration when receiving treatment due to medical, physical, cognitive, or developmental conditions [9]. The population includes children with behavior (eg, autism spectrum, anxiety, attention deficit hyperactivity disorder), congenital (eg, trisomy 21, congenital heart disease), developmental (eg, cerebral palsy), systemic (eg, childhood cancer, sickle cell disease), or cognitive disorders (eg, intellectual disability) [9]. Children with SHCN face more barriers to dental care than the overall population [9]. Barriers include external factors (transportation, cost, inadequate dental facilities) and internal factors (fear and poor tolerance) [9,10]. Many children with SHCN are cared for by dentists in the community or, more often, in hospital dental clinic settings [9]. They experience more DFA, which can result in more difficult dental visits [9]. The importance of providing well-rounded care and making each dental visit a positive one for pediatric patients with SHCN is crucial for promoting a good oral health routine, as well as improving their oral health transitioning into adulthood [9].

Understanding and assessing DFA in children are important for delivering successful dental care with high satisfaction in this age group. Among the vast number of assessment method options available today, self-report assessment, parental proxy assessment, observation-based assessment, and physiological assessment are the 4 major types for DFA in children [11]. In the recent literature, salivary alpha-amylase, an enzyme correlated with both adrenaline and noradrenaline, has also been used as a marker for autonomic nervous system activity and stress [12].

Factors in the dental setting that trigger DFA include the loud sounds of dental instruments, presence of strangers examining the oral cavity, and the fear of pain [13]. Of these, the biggest trigger of DFA is the anticipation and use of local anesthesia injections [5,13]. Although necessary to provide adequate pain control during certain treatments, local anesthesia injections are understandably uncomfortable [5]. For children, the initial injection combined with the feeling of numbness during the procedure is especially distressing [5]. Pharmacological agents, combined with light to moderate sedation, or general anesthesia can also be considered for noncooperative patients; these options are often time-consuming and confer a higher cost as well as health risk [1]. In the current literature, audiovisual distractions such as tablets or TV screens have been used as additional distraction techniques rather than just traditional tell-show-do distraction, with overall positive results [14]. However, there is a lack of interactivity with these techniques, and as a result, they produce less immersive environments with distraction for the children [15]. Lack of patient cooperation due to DFA often obliges dentists treating pediatric populations to end appointments prematurely and sometimes without completing the planned procedure. Treating an anxious and fearful patient can create an environment of stress for the clinician and associated dental team [1].

Particularly when treating children with SHCN, extra time and tools are needed to provide comfortable dental care. Depending on the child’s diagnosis, some have hypersensitivity to external stimuli such as loud noises, aversion to specific tastes, and difficultly straying from usual daily routines [16]. A study by Pagano et al [16] showed that the use of augmented reality was well suited for patients with autism spectrum disorder in preparation for their dental visits. Additionally, a systematic review by Cunningham et al [17] concluded that virtual reality (VR) is a promising tool in dentistry, especially for the population of children with autism spectrum disorder or other special needs.

VR is defined as an artificial environment that is experienced through sensory stimuli [18]. It is a modern tool that can immerse patients in a “game” or “world.” Commonly used in the medical field to help distract patients during unpleasant procedures such as vaccination, cast removal, and short bedside interventions, it has proven to be effective at decreasing procedural anxiety and providing a more positive experience for patients [19]. Among the limited existing literature, the use of VR to manage anxiety during dental procedures has shown positive results. A recent clinical trial by Alshatrat et al [15] concluded that VR is an effective tool for reducing anxiety in young children during dental procedures. Moreover, a previous study by Ram et al [20] showed that both parent and clinician satisfaction was high using audiovisual glasses as distraction for children during dental treatments. However, clinical VR research in pediatric dentistry is limited, especially with special needs populations. VR use in pediatric dentistry offers the potential to add a nonpharmacologic tool to a clinician’s toolbox. A clinical study on the use of VR during dental appointments with pediatric patients with SHCN would allow a better understanding of the effect of VR on DFA in this population and possibly facilitate dental procedures.

### Aims of the Study

The aims of the study are twofold: (1) assess the feasibility and acceptability of VR immersion as a tool to reduce DFA in pediatric patients with SHCN undergoing dental procedures and (2) gain insight on parent and health care provider perspectives on the use of VR during dental appointments.

### Objectives

The primary research objectives are to determine (1) the feasibility and acceptability of VR distraction for children with special needs requiring dental procedures, (2) compare parent and health care professional satisfaction levels between the VR distraction group and the clinic’s standard mounted TV showing cartoons, and (3) observe the preliminary effects of VR distraction on reducing patient anxiety during dental procedures compared with standard nonpharmacological behavior management.

The secondary objectives of this study are to compare the following between the VR distraction group and the clinic’s standard mounted TV showing cartoons: (1) physiological parameters (pulse and oxygen saturation), (2) the occurrence of side effects, (3) dental procedure length, (4) the number of retakes of dental procedures due to DFA, and (5) salivary amylase levels.

## Methods

### Design

This randomized controlled trial pilot study will follow a parallel design including 2 groups: a control group (mounted wall TV playing cartoons) and an experimental group (VR intervention during the dental procedure).

### Sample and Setting

This pilot will include 20 participants, totalling 10% of the expected 200 children to be included in the final study. All participants in this pilot study will be allocated in an equal ratio of 10 per group. Recruitment will be carried out at the dental clinic of the Centre Hospitalier Universitaire Sainte-Justine, a pediatric hospital in Montreal, Canada. This clinic mostly serves patients with SHCN such as craniofacial abnormalities, autism spectrum disorder, cancer, and others. Pediatric patients with SHCN represent around 80% of the total clientele of this clinic, while the rest is comprised of otherwise healthy patients with dental traumas and other dental emergencies.

### Inclusion Criteria

Children participating in this study will have to meet the following criteria: (1) aged 6 years to 17 years; (2) recommended by the dentist to participate; (3) required to undergo any dental procedure; and (4) accompanied by a parent or a legal guardian who can understand, read, and write in either French or English.

### Exclusion Criteria

Participants will be excluded from this study if they meet the following criterion: have epilepsy or any other conditions preventing them from using VR. Non-SHCN patients will not be excluded from the study, but this information will be collected. Patients with a strong history of motion sickness will not be excluded from this study, but this information will be collected and additional monitoring will be provided. The use of medication (opioid and non-opioid analgesic, antiemetic, anxiolytic, or any other drugs) prior to the procedure within the last 4 hours will not exclude participants from this study, but this information will be collected during the pre-intervention questionnaire. Patients having undergone previous dental procedures in the past will not be excluded.

### Randomization and Allocation

Randomization will be done through the electronic REDCap (Vanderbilt University) system. Allocation to either intervention will be randomized by an independent biostatistician from the Applied Clinical Research Unit (URCA). To equalize participants in both arms, permuted block randomization with a randomly selected block size design will be used to randomize participants to the intervention [21]. Access to the randomization list will only be granted to the biostatistician, and allocation will be concealed using REDCap to reduce selection bias.

### Interventions

#### Control Treatment

The control group will only receive a care-as-usual approach. This includes a television mounted on the wall showing cartoons and the use of pharmaceutical treatment during the procedure such as the use of injected local anesthesia. In the event of noncooperation during the appointment, any retakes or rescheduling of appointments will be compiled by the resident dentist. One parent will be permitted to be present in the room during the procedure as part of the clinic’s usual protocol, and their presence will be recorded. Children allocated to the control group will be offered the possibility to try the VR game after the study period if they choose to do so.

#### Experimental Treatment

The experimental group will be provided the VR video game “Dental Dream” designed specifically for this study. Pharmaceutical treatment, such as the use of injected local anesthesia, during the procedure will be used if the procedure requires it. Children will be able to play for the entire duration of the dental procedure. “Dental Dream” aims to reduce anxiety in children aged 6 years to 17 years old by means of immersive distraction. The VR headset offers children the ability of viewing the game they are playing in real time while simultaneously obstructing the partial view they would normally have of the procedure. In the event of noncooperation during the appointment, any retakes or rescheduling of appointments will be compiled by the resident dentist. One parent will be allowed in the room during the dental procedure, and their presence will be recorded.

Developed by Paperplane Therapeutics, “Dental Dream” is an easy-to-play, immersive VR video game tailored for the pediatric population and approved by a team of health care professionals in pediatric care. The simplified no-success game allows it to be enjoyable no matter the child’s video game experience, and its point-and-shoot arcade style also allows for easy understanding and fast immersion in the game. Positioning is normally a challenge for VR as it is very position-specific, needing a semi-fowler's angle of 30°-45° to function properly. “Dental Dream” was designed specifically for this study with the specific horizontal position needed for dental procedures in mind. “Dental Dream” is designed to be supported by the Pico Neo 4 VR headset.

Children playing the game will be immersed through a virtual environment where they will be able to use a remote in one hand to target floating and static objects in the VR game. The objective of the game is to throw balls at targets such as balloons, trolls, and diamonds to gain points. The “Dental Dream” game uses an on-rail feature guiding the child through space with a new eye tracking technology developed within the headset in order to help the child navigate the same way head movement normally would in classic VR, making it easier for dental procedures where head movement is restricted. These features also aim to reduce cybersickness.

The preliminary version of the VR headset has been tested in clinic on staff—the size and volume of the Pico Neo 4 headset do not hinder delivery of dental care as it is smaller than usual VR headsets.

### Study Time Points

Sociodemographic data will be assessed before the intervention at baseline (T0) to establish a baseline using a simple questionnaire completed by the parent or legal guardian present while sitting in the waiting room. Salivary alpha-amylase samples will be collected at T0 and after the intervention/procedure (T2; Multimedia Appendix 1). Measures of specific dental procedure–related anxiety and uncooperative behavior will be assessed at T0, during the procedure (10 minutes from the start; T1), and immediately after the end of the procedure (T2). Physiological parameters will be measured at T0, during the intervention (T1), and T2. Data on occurrence of side effects will be assessed and monitored at all times for the duration of this study. All clinical monitoring will be performed by the dental resident on site. Measures of parent or legal guardian and health care professional satisfaction will be assessed at T2.

### Measures and Outcomes

#### Sociodemographic Characteristics

Sociodemographic characteristics will be provided by the parent or legal guardian in the waiting room prior to the intervention and will include information such as age, sex, ethnicity, and the procedure needed. Other information will also include any medication taken within the last 24 hours (name, class, and posology) that could have an impact on the conclusions of this study.

#### Primary Outcomes

To measure feasibility and patient acceptability, we will record patient recruitment rates, completion rates of planned procedures, and satisfaction. As this is a population of children with SHCN, we estimate 50% of patients will agree to participate during recruitment, and we expect a completion rate of planned procedures of 75%.

#### Measures of Primary Outcomes

Parent or legal guardian satisfaction with the intervention will be assessed using a visual analog scale (VAS; 0-10, where 0 is very dissatisfied and 10 is very satisfied) and the question recommended by the Pediatric Initiatives on Methods, Measurement and Pain Assessment in Clinical Trials (PedIMMPACT): “Considering anxiety relief, side effects and emotional recovery, how satisfied were you with the intervention used to manage DFA experienced by your child?” [22] (Multimedia Appendix 2). The parent or legal guardian will respond according to their satisfaction level.

Health care professional satisfaction with the intervention will be assessed using a VAS (ranked from 0-10, where 0 is very dissatisfied and 10 is very satisfied) as well as a 6-question satisfaction questionnaire (each answer will be given a score of 1, 2, 3, or 4 based on the choice of answer: Fortement en désaccord, En désaccord, En accord, Fortement en accord, respectively) and will result in a final score between 6 and 24 (Multimedia Appendix 3).

DFA scores will be evaluated using the Venham Anxiety and Behavioral Rating Scale [23]. It is an observation-based assessment by proxy for DFA and is among the most frequent behavior scoring models for DFA [23] (Multimedia Appendix 4). It is composed of 2 scales used to assess the anxiety and uncooperative behavior of children in the dental setting. The scale is also found under the name Clinical Anxiety Scale/Uncooperative Behaviour Rating Scale or Venham Clinical Cooperation Scale and Venham Clinical Anxiety Scale and has been used as an anxiety rating scale in other studies that have evaluated the efficacy of VR distraction for the management of DFA [11,24,25]. Both consist of a 6-point scale, with 6 defined behavioral levels that range from 0 to 5. The highest score defines the highest anxiety level or lack of cooperation [23]. A high degree of reliability has been observed for both scales, even for an untrained observer [23,26]. The scale points are measured by proxy and anchored in objective, specific, and readily observable behavior. The research assistant indicates the participant’s behavior by picking a number from 0 to 5 according to the scale; thus, for each participant, 2 scores on the levels of anxiety and cooperation with the dental procedure will be obtained at T0, T1, and T2. Venham et al [27] reported that the scale is a reliable and valid scale and provides an interval-level measurement. The scale has been a useful instrument for assessing a child’s response to dental stress. The anxiety score and uncooperative score will both be evaluated at T0 (baseline), during the procedure at 10 minutes after the beginning of the procedure (T1), and after the completion of the procedure (T2). The videos will be reviewed by 2 independent evaluators who will provide a score for the anxiety of each child during the procedure. Scores will be compared afterwards. The same scales will be used during observation of the child’s behavior using the video recordings by a separate member of the team. Both evaluations (when available depending on consent) will allow for inter-rater agreement to be calculated.

#### Secondary Outcomes

The secondary outcomes will be (1) changes in physiological parameters (such as heart rate and pulse oximetry) during and after the intervention compared with baseline, (2) occurrence of side effects, (3) deviation from the normal procedure length, (4) rescheduling of procedures in the event cooperation is impossible, and (5) salivary amylase levels.

#### Measures of Secondary Outcomes

The secondary outcomes will be measured as described here. Physiological parameters will be continuously measured using the Nellcor pulse oxygen saturation meter (Covidien), an approved and validated device. The occurrence of side effects will be collected from arrival on site to discharge from the study using a checklist of common side effects experienced while using VR and also related to dental medication. The length of the procedure will be measured and collected for every participant and will be compared with the average duration for a similar procedure that will have been measured prior to the study by the clinic’s personnel. Rescheduling of procedures in the event that cooperation is impossible will be noted. Salivary amylase levels, as a stress biomarker, will be measured before and after the dental procedure as described in the following section.

#### Measurement of Salivary Amylase

Saliva samples will be collected from all children in this study at 2 different study time periods: before (T0) and after (T2) the intervention. Salivary alpha-amylase has been recognized and validated as a reliable and specific marker for autonomic nervous system activity and has been proposed as an alternative biomarker for stress [12]. Alpha-amylase sampling has been performed in previous studies to evaluate DFA for children undergoing dental procedures [28-30]. Alpha-amylase, as a salivary biomarker, can be considered an important and noninvasive tool for assessment of anxiety-related events, such as dental extraction and other treatment, for children [28,31].

Salivary samples will be collected using a sterile dental cotton swab from under the tongue 10 minutes before and 10 minutes after the procedure and stored immediately within a sterile vile in a research-specific on-site freezer. Samples will be collected by the dental resident and taken to the laboratory. Results will be entered in REDCap for analysis after the study.

Table 1 provides an overview of which questionnaires will be answered by which respondents during the study.

**Table 1:** Questionnaires and corresponding respondents.

Questionnaires	Respondents
Parent or legal guardian satisfaction	Parents or legal guardians
Health care professional satisfaction	Health care professional
Anxiety score, physiological parameters	By proxy through research assistants

### Study Proceedings Including Data Collection

Participants will be identified by the resident dentist through the appointment scheduling system as requiring any dental procedure. Since DFA can arise from multiple different procedures such as simple cleaning to teeth extraction, dentists at the clinic will identify possible participants to include based on their cooperation, personality, and fears. An individual independent to the study will review consent with participants and parents. Written consent by the parents or legal guardian—including the child’s assent—will be obtained on arrival at the clinic the day of the procedure while completing the preprocedural questionnaire.

After verifying that the participant meets all the inclusion and exclusion criteria, the dental resident will log into REDCap only 10 minutes before the start of the intervention to minimize the risk of bias toward the intervention and to obtain the group allocation and announce it to the participant. Because of the nature of VR, no blinding of staff, participants, and parents or legal guardians will be possible. Baseline data collection prior to the dental procedure, including the sociodemographic questionnaire, recording of physiological parameters, and salivary alpha-amylase sampling, will take approximately 15 minutes to complete. The VR headset will be adjusted for the child’s size, and approximately 5 minutes will be allotted for children to familiarize themselves with the room, equipment, and game before the start of the procedure.

The intervention will last the entire time of the dental procedure, and the duration will be recorded for every patient. DFA assessment by proxy using the Venham Anxiety and Behavioral Rating Scale will be performed on site by the dental resident present and will also be filmed (if consent was given) to be evaluated by another member of the team at a later date.

As per the clinic’s protocol, if a child becomes restless and cooperation is impossible, the child will be held by his or her parents for the remaining duration of the procedure if it cannot be safely stopped at that time or if the procedure is considered an emergency. If the procedure can be safely stopped, rescheduling will be discussed with the parents including the possible need for sedation. The involvement of parents will be recorded as well as any other distractions used during treatment such as the use of stickers, teddy bears, or music.

All side effects and medication used will be recorded. The former will be transmitted to the ethics committee if needed. As VR has the potential to cause nausea, dizziness, and motion sickness, should participants experience any of these, they will be dealt with according to the clinic’s protocols. If participants experience adverse side effects and the attending dentist and dental assistant have been informed, the treatment will be safely discontinued. All instruments in the mouth will be removed. The VR device will be removed from the participant. The dental chair will be placed in a comfortable position, and the patient will rest until they feel comfortable and recovered. The option of continuing the treatment (without the VR headset) or postponing the treatment to another day will be discussed. Postprocedure salivary alpha-amylase sampling and completion of questionnaires regarding parent and health care professional satisfaction levels after the dental procedure will take approximately 20 minutes. Equipment used by children will be disinfected before the next participant arrives.

### Statistical Analysis

Analysis will be conducted using the SAS (SAS Institute) statistical analysis software (version 9.4). Descriptive statistics, by treatment, will be conducted for demographic and clinical variables and will be used to present sociodemographic and clinical data, parent and health care professional satisfaction levels, and procedural time. Due to a small sample size, we will also assess effect sizes.

For the pilot project, we plan to perform descriptive analyses of the sample as well as comparative analyses between the 2 groups. Nonparametric tests for procedural type, age, parental presence, parental and health care satisfaction levels, and baseline (T0) anxiety score measurement will be used to assess the composite outcome for mean salivary cortisol level and mean difference in anxiety score (using the Venham Clinical Cooperation Scale and Venham Clinical Anxiety Scale) between the experimental and control groups at T2. Analyses will be carried out according to the intention-to-treat principle, with a significance level (α) of .025, considering Bonferroni's correction.

For secondary outcome analyses, nonparametric tests adjusted for procedural type, age, and parental presence will be used to assess the mean procedural length at T2 using the VAS and 6-item tailored questionnaire, respectively. Comparison of dichotomous variables including the necessity for procedural retakes, use of other nonpharmacological interventions, and the occurrence of side effects will be assessed using the Cochran-Mantel-Haenszel test.

The study data will be recorded in the REDCap system belonging to URCA. The data manager associated with URCA and independent of the study will be responsible for the storage of the recorded data. However, a URCA biostatistician independent of the study will be responsible for the data analysis. The data analysis plan will be discussed with the principal investigator (SLM). Interjudge correlations will be conducted for the video tape analysis. Two judges will independently rate the anxiety measure on the videotapes. Subsequently, we will conduct intraclass correlation coefficient analyses to compare the scores for each of the recruited patients between the 2 judges.

### Ethical Considerations

Approval by the Ste-Justine Hospital research board of ethics (#2023-4985) was obtained in March 2023. An individual independent to the study will review consent with participants and parents. The information and consent form will be signed by one of the parents on the day of the visit. Upon enrollment, children are assigned codes used on all data collection forms to protect their confidentiality. Only the dental resident and the principal investigator will have access to the log that links the study codes to the participants. Patient-identifying information is kept separate from the case reports and is linked to study enrollment by a study ID number. The consent form also identifies all the information that may be collected during this study and details the use of videos that may be needed for further behavior evaluation using proxy scales with the possibility to opt out. All data collected by paper forms the day of the intervention will subsequently be transferred into REDCap. No recording of the child’s screen nor their game inputs will be collected. The intervention will be filmed and recorded, as the use of proxy scales will need further evaluation of the child’s behavior. As per usual, all information such as links between patient identifiers to study ID and paper copies of forms and questionnaires will be kept stored and double locked for 7 years (starting from the end of trial) at the principal investigator’s office at the research center.

## Results

This study will be conducted from May 2023 to May 2024, with results expected to be available in December 2024. The findings of the study will be disseminated through peer-reviewed publications.

## Discussion

The results of the study will allow us to gain insight on the feasibility and acceptability of the intervention and on the satisfaction of parents and health care professionals regarding the use of VR as a tool to reduce anxiety in pediatric dentistry, especially in children with SHCN. Due to the limited clinical studies about VR and pediatric SHCN populations in dentistry, this pilot study will guide potential future, larger, clinical trials.

### Challenges and Limitations

Individual experience with VR varies immensely, as some children may love to have an active distraction during dental treatment, while others may prefer to be present in the moment. As such, we acknowledge that the acceptance of VR use during dental treatment is a tool that may be useful for a certain population within a patient pool. Like all other distraction tools used in the dental setting, proper screening and patient selection are crucial during treatment planning. Moreover, the game design used during the study was created specifically for our study, so the results of our study may not be able to be extrapolated to a different setting with a different game design.

### Conclusion

The results of the study could be used to help us to better understand how VR distraction can reduce DFA in children with special needs and the acceptability of its use by parents, patients, and health care professionals during dental procedures. The results can be used as preliminary data for a future clinical trial on the use of VR in dentistry with special needs patients. In addition, this study will possibly allow for more comfortable and less traumatic dental care for pediatric patients. By exploring new avenues for behavior and anxiety management tools for pediatric dentistry, this study and its results will provide improved patient-centered care.

## Appendix

Multimedia Appendix 1 Study time points and data collection.

Multimedia Appendix 2 Parental satisfaction questionnaire.

Multimedia Appendix 3 Healthcare professional satisfaction questionnaire.

Multimedia Appendix 4 Venham anxiety and behavioral rating scale.

## Abbreviations

DFA: dental fear and anxiety
SHCN: special health care needs
URCA: Applied Clinical Research Unit
VAS: visual analog scale
VR: virtual reality

## Data Availability

Once the pilot study is completed, the authors confirm that the data supporting the findings of this study will be available through the clinicaltrials.gov repository. In addition, data will be available within a future publication and its supplementary materials.
