# Supplementary material for: Effects of a Virtual Reality Game on Children’s Anxiety During Dental Procedures (VR-TOOTH): Protocol for a Pilot Randomized Controlled Trial
Source: JMIR Res Protoc. 2023 Nov 10;12:e49956. doi: 10.2196/49956 (PMC10674143; doi:10.2196/49956)
Supplement: Multimedia Appendix 1 [file resprot_v12i1e49956_app1.docx]

**APPENDIX 1: Study time points and data collection**

| T0:  Baseline | 1. Sociodemographic questionnaire 2. Venham’s Clinical and Behavioral scores 3. Alpha-amylase sampling 4. Physiological parameters* |
| --- | --- |
| T1:  10 minutes after beginning of dental procedure | 1. Venham’s Clinical and Behavioral scores 2. Physiological parameters* |
| T2:  After the end of dental procedure | 1. Venham’s Clinical and Behavioral scores 2. Physiological parameters* 3. Parent and healthcare professional’s level of satisfaction 4. Alpha-amylase sampling |

*Physiological parameters include heart rate and pulse oximeter
