# Supplementary material for: Effects of a Virtual Reality Game on Children’s Anxiety During Dental Procedures (VR-TOOTH): Protocol for a Pilot Randomized Controlled Trial
Source: JMIR Res Protoc. 2023 Nov 10;12:e49956. doi: 10.2196/49956 (PMC10674143; doi:10.2196/49956)
Supplement: Multimedia Appendix 2 [file resprot_v12i1e49956_app2.docx]

**APPENDIX 2**

**Échelle de satisfaction parent/tuteur:**

En tenant compte de la prise en charge de l’anxiété, des effets secondaires et du rétablissement émotionnel, êtes-vous satisfait des traitements que votre enfant a reçu pour la gestion de l’anxiété relié à la procédure dentaire?

*Considering anxiety relief, side effects and emotional recovery, how satisfied were you with the intervention used to manage dental fear and anxiety experienced by your child?*


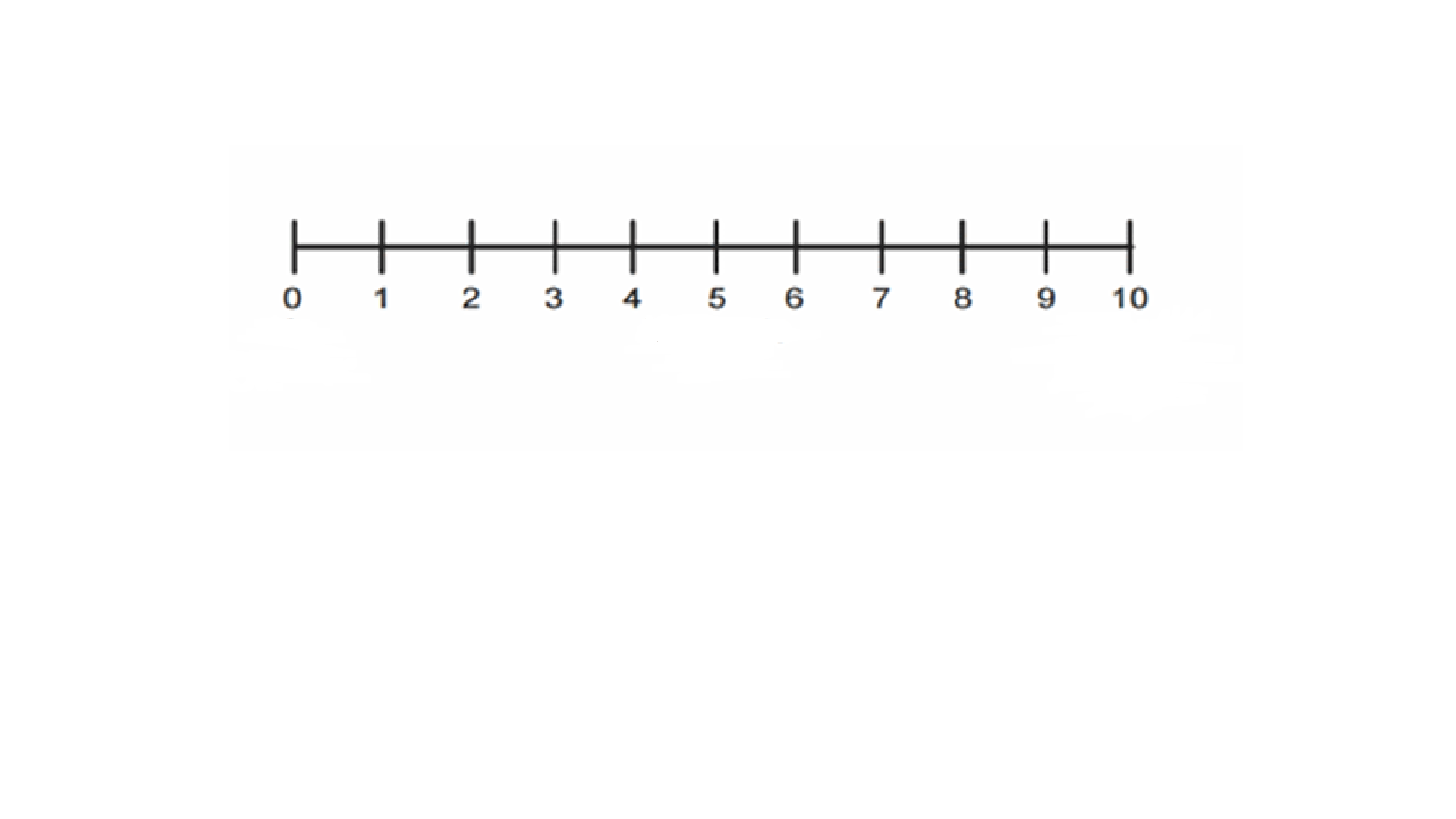


Score: ______/10 (auto-évaluation)

Avez-vous des commentaires? : ____________________________________________________________________________________________________________________________________________________________________________________________________________________________________________________________________________________________________________________________________________________
