# Supplementary material for: Effects of a Virtual Reality Game on Children’s Anxiety During Dental Procedures (VR-TOOTH): Protocol for a Pilot Randomized Controlled Trial
Source: JMIR Res Protoc. 2023 Nov 10;12:e49956. doi: 10.2196/49956 (PMC10674143; doi:10.2196/49956)
Supplement: Multimedia Appendix 3 [file resprot_v12i1e49956_app3.docx]

**APPENDIX 3**

| **Projet de recherche :** La réalité virtuelle pour la gestion de l’anxiété procédurale lié aux procédures dentaires chez les enfants ayant des besoins particuliers en matière de soins de santé : Une étude pilote contrôlé randomisé.  Vous êtes invité à compléter ce questionnaire de satisfaction si vous avez effectué une ou plusieurs procédures dentaires chez un ou plusieurs enfant(s)/adolescent(s) ayant accepté de participer à l’étude dont **l’objectif est de comparer la réalité virtuelle à la prise en charge standard pour la gestion de l'anxiété procédurale lié aux procédures dentaires.**  Nous aimerions connaître votre niveau de satisfaction par rapport à ces deux interventions. Pour chacune de ces deux interventions, veuillez cocher la case qui représente le plus votre niveau de satisfaction. Si vous n’avez pas eu l’occasion d’utiliser l’une ou l’autre des interventions, veuillez l’indiquer à l’endroit échéant.  La participation à cette étude est libre et volontaire. Vous êtes donc libre de refuser de remplir ce questionnaire de satisfaction. Si vous acceptez de remplir ce questionnaire, l’ensemble des informations recueillies demeureront confidentielles. En remplissant ce questionnaire, vous consentez à l’étude. |
| --- |

| **Questionnaire sur la satisfaction des intervenants** |
| --- |

**Titre professionnel :**

Infirmier/Infirmière

Assistant(e) dentaire

Dentiste

Dentiste en formation (Résident)

Autre: .............................

**Intervention : Réalité virtuelle**

**Avez-vous assisté à une procédure durant laquelle la réalité virtuelle a été utilisée?**

**Oui ☐ Non ☐**

**Si non, passez à l'intervention suivante, si oui, combien de fois?**

**Moins de 5 fois ☐ Plus de 5 fois ☐**

| **Énoncés** | **Fortement en désaccord** | **En désaccord** | **En accord** | **Fortement en accord** |
| --- | --- | --- | --- | --- |
| **1.** La réalité virtuelle a aidé l’enfant à contrôler son anxiété/peur |  |  |  |  |
| **2.** La réalité virtuelle a nui au déroulement de la procédure |  |  |  |  |
| **3.** La réalité virtuelle a permis à l’enfant de mieux coopérer durant la procédure |  |  |  |  |
| **4.** Je réutiliserais la réalité virtuelle pour d’autres procédures dentaires chez les enfants |  |  |  |  |
| **5.** Le dispositif de réalité virtuelle était adapté à l’environnement de la salle de procédures |  |  |  |  |
| **6.** Le concept de l’utilisation de la réalité virtuelle lors des procédures dentaire est une idée qui vaut la peine d’être développée |  |  |  |  |

| **Votre préférence :**  **Réalité virtuelle**  **Distraction passive** |
| --- |

**COMMENTAIRES ET AMÉLIORATIONS À APPORTER :**

**MERCI DE VOTRE COLLABORATION!**
