# Supplementary material for: Effects of a Virtual Reality Game on Children’s Anxiety During Dental Procedures (VR-TOOTH): Protocol for a Pilot Randomized Controlled Trial
Source: JMIR Res Protoc. 2023 Nov 10;12:e49956. doi: 10.2196/49956 (PMC10674143; doi:10.2196/49956)
Supplement: Multimedia Appendix 4 [file resprot_v12i1e49956_app4.docx]

**APPENDIX 4**

**Venham Anxiety and Behavioral rating scale (Venham et al. 1980)**

| Venham Anxiety Rating Scale (VARS)* | |
| --- | --- |
| 0 | Relaxed, smiling, willing, and able to converse |
| 1 | Uneasy, concerned. During stressful procedure may protest briefly and quietly to indicate discomfort. Hands remain down or partially raised to signal discomfort. Child willing and able to interpret experience as requested. Tense facial expression, may have tears in eyes |
| 2 | Child appears scared. Tone of voice, questions and answers reflect anxiety. During stressful procedure, verbal protest, (quiet) crying, hands tense and raised, (not interfering much may touch dentist’s hand or instrument, but not pull at it). Child interprets situation with reasonable accuracy and continues to work to cope with his/her anxiety |
| 3 | Shows reluctance to enter situation, difficulty in correctly assessing situational threat. Pronounced verbal protest, crying. Using hands to try to stop procedure. Protest out of proportion to threat. Copes with situation with great reluctance |
| 4 | Anxiety interferes with ability to assess situation. General crying not related to treatment. More prominent body movement. Child can be reached through verbal communication, and eventually with reluctance and great effort he or she begins the work of coping with the threat |
| 5 | Child out of contact with the reality of the threat. Genera1 loud crying, unable to listen to verbal communication, makes no effort to cope with threat. Actively involved in escape behavior. Physical restraint required |

*Other name: Venham Clinical Anxiety Scale (VCAS) (Sahebalam et al. 2020)Clinical Anxiety Rating Scale (Yon et al. 2020)

| Venham Behavioral Rating Scale (VBRS)** | |
| --- | --- |
| 0 | Total cooperation, best possible working conditions, no crying or physical protest |
| 1 | Mild, soft verbal protest or (quite) crying as a signal of discomfort, but does not obstruct progress. Appropriate behavior for procedure |
| 2 | Protest more prominent. Both crying and hand signals. May move head around making it hard to administer treatment. Protest more distracting and troublesome. However, child still complies with request to cooperate |
| 3 | Protest presents real problem to dentist. Complies with demands reluctantly, requiring extra effort by dentist. Body movement |
| 4 | Protest disrupts procedure, requires that all of the dentist attention be directed toward the child behavior. Compliance eventually achieved after considerable effort by dentist, but without much actual physical restraints. More prominent body movement |
| 5 | General protest, no compliance or cooperation. Physical restraint is required |

**Other name: Venham Clinical Cooperation Scale (VCCS) (Sahebalam et al. 2020) or Uncooperative Behaviour Rating Scale (Yon et al. 2020)
